# Advanced molecular tools for surveillance and management of tobamoviruses

**DOI:** 10.3389/fpls.2025.1718133

**Published:** 2026-01-14

**Authors:** Mirza Abid Mehmood, Muhammad Mazhar Iqbal, Muhammad Ashfaq, Suiyun Chen, Jianguang Wang

**Affiliations:** 1 State Key Laboratory of Vegetation Structure, Function and Construction, Ministry of Education Key Laboratory for Transboundary Ecosecurity of Southwest China, Crop Disease & Pest Biocontrol Engineering Research Center of Yunnan Province, School of Ecology and Environmental Science, Yunnan University, Kunming, Yunnan, China; 2 Plant Pathology, Institute of Plant Protection, Muhammad Nawaz Shareef University of Agriculture, Multan, Pakistan

**Keywords:** CRiSPR/Cas, genome editing, omics approaches, RNA interference (RNAi), tobamovirus resistance, ToBRFV

## Abstract

Tobamoviruses are a group of plant viruses that can cause yield losses of up to 70% and reduce fruit quality by 30–50%. Historically, tobamoviruses were dominated by tobacco mosaic virus (TMV) and tomato mosaic virus (ToMV). However, the landscape is rapidly shifting with the emergence of economically significant viruses such as tomato mottle mosaic virus (ToMMV) and tomato brown rugose fruit virus (ToBRFV). Both can circumvent the previously durable Tm-2² resistance in tomato and spread across multiple continents. This shift coincides with dramatic leaps in diagnostic tools, which have enhanced surveillance capabilities. Sensitive detection of tobamoviruses in the field with minimal sample preparation can be achieved using latest technologies such as isothermal amplification, CRISPR/Cas-hybrid assays or next-generation sequencing. Virus-host interactions underscore that viral proteins, including replicase components, are potent suppressors of RNA silencing (VSRs). Small RNA profiling and network analyses of viral movement proteins reveal complex mechanisms of immune evasion and resistance breakdown. These findings are largely based on dominant NB-LRR genes such as *L*, *Tm-1*, and *Tm-2^2^*. However, evidence indicates that ToBRFV can bypass this resistance via mutation in the movement protein, so supplementary methods should be considered. This review covers latest approaches, such as genome editing with CRISPR, targeting susceptibility genes, RNA interference (RNAi), and multi-omics approaches (transcriptomics, proteomics, metabolomics, ionomics), that can facilitate real-time surveillance and breeding for enhanced resilience. Moreover, the use of bio-formulations and nano-formulations as eco-friendly alternatives against tobamoviruses is discussed in detail. Climate change further complicates disease dynamics by undermining temperature-sensitive resistance, altering virus prevalence, and exacerbating yield losses. The rapid emergence of new tobamoviruses, which threatens the economy, necessitates a comprehensive approach. The integration of molecular diagnostics using CRISPR, omics technologies, designed protective systems, and climate-augmented disease prediction offers a detailed blueprint for the sustainable control of tobamoviruses and crop protection.

## 1 Introduction

Tobamoviruses (*Tobamovirus, Virgaviridae*) are RNA viruses with ~ 6.4 kb single-stranded genomes, encapsidated in rigid rod virions that aid their survival during mechanical transmission. They can persist on surfaces, in soil, and spread via seed transmission. These viruses disseminate rapidly through direct contact and contaminated propagative materials (Zhang et al., 2022). Historically, tobacco mosaic virus (TMV) and tomato mosaic virus (ToMV) were significant disease-causing agents, but the introduction of resistance genes like *Tm-2²* has mitigated their impact. Species resulting from horizontal gene transfer, such as tomato mottle mosaic virus (ToMMV) and tomato brown rugose fruit virus (ToBRFV), present a new challenge to global solanaceous crop yields. ToBRFV, first identified in Jordan and Israel in 2015, has since caused devastating outbreaks in greenhouse tomato crops. It is reported to infect over 90% of plants and lead to losses as high as 70% (Caruso et al., 2022; Salem et al., 2023). Importantly, ToBRFV also breaks the Tm-2² resistance gene, leading to quarantine measures across Europe, North America, and Asia. Additionally, tobamoviruses are considered as quarantine pests because of their remarkable stability, ease of transmission, and their association with severe yield and quality losses in solanaceous and cucurbit crops. Tobamoviruses are under the European and Mediterranean Plant Protection Organization (EPPO) A2 quarantine list (EPPO A2 List, 2025). Consequently, strict phytosanitary measures, seed certifications, and import regulations have been implemented worldwide, to prevent or minimize their transboundary movements and establishment.

The increasing global spread of tobamoviruses is a matter of concern. Originally detected in 2013 in Mexico, ToMMV is now globally distributed in commercial seed lots and field samples. It frequently co-infects with ToMV and ToBRFV, necessitating integrated and globally multimodal surveillance (Chanda et al., 2021). Understanding virus evolution, seed transmission pathways, diagnostic innovations, and host-pathogen interactions is essential for protecting tomato and pepper production in a highly connected and climate-vulnerable agricultural landscape. Surveillance of emerging tobamoviruses requires advanced diagnostic technologies. Multiplex and duplex RT-qPCR methods have simultaneously detected ToBRFV and ToMMV in both leaf and seed samples with high sensitivity and reproducibility. Their detection capacity is as low as 1 infected seed in 800 non-infected seeds (Chanda et al., 2021). In 2021, RT-LAMP-based assays were developed, enabling sensitive commercial RT-LAMP kits with a reported limit of detection above 1 g/mL, and 24 kits have a quoted performance range (Rizzo et al., 2021). CRISPR/Cas12a-based diagnostics targeting the ToBRFV movement protein gene have been developed, demonstrating species-specific detection (no cross-reaction with TMV, ToMV, PMMoV, TMGMV), visual readout capability, and reliable detection comparable to RT-qPCR (Bernabé-Orts et al., 2022). Recent studies suggest further investigation of molecular and technological aspects of tobamovirus research. Multi-omics approaches (e.g., transcriptomics, proteomics, and metabolomics) have not been widely implemented to investigate host–virus interactions; molecular signaling, systemic defense mechanisms, and plant responses to tobamoviruses in a range of crops (Satrio et al., 2024). While relevant studies have shown practical advances towards understanding other plant-pathogen systems, the full potential and implications of utilizing such studies towards tobamovirus biology have yet to be fully realized. Similarly, the incorporation of new diagnostics (e.g., CRISPR-based biosensors), portable sequencing platforms (e.g., Oxford Nanopore), and high-throughput applications for serological assays diagnostics have rarely been adopted for rapid and soil-based diagnosing of tobamoviruses (Suman et al., 2024). This review elaborates the use of molecular diagnostics and technological approaches, incorporated into future risk assessments, represents a logical step to begin estimating relationships of tobamovirus evolution and epidemiology, host adaptation to infectivity, and management practices.

## 2 Historical background, global distribution, and host range of tobamoviruses

The historical perspective of tobamoviruses plays a key role in exploring the history of plant viruses. TMV, a prototypical member of this genus, was first described in late 19^th^ century when scientists observed the mosaic type patterns on tobacco leaves (Carr, 2004). This condition had a considerable economic impact on tobacco yield. TMV remained a model organism for research in molecular biology throughout the 20^th^ century. The study of tobamoviruses extended beyond TMV, encompassing other important plant viruses like ToMV and Pepper mild mottle virus (PMMoV). Some of the other major tobamoviruses along with their global distribution are given in **Table 1**.

**Table 1:** Global distribution of major tobamoviruses.

Tobamovirus	Countries	Year of first report	Host range	Reference
Tobacco mosaic virus (TMV)	Global distribution; first identified on tobacco plants (initially in USA and then in Europe)	Early 20th century	Solanaceae, Brassicaceae, Cucurbitaceae, Malvaceae, Asteraceae, Apocynaceae, Leguminosae, Passifloraceae, Cactaceae, Orchidaceae, Scrophulariaceae, Gesneriaceae	(Ishibashi et al., 2023)
Tomato mosaic virus (ToMV)	Globally distributed, initially reported in tomato-growing areas of Europe	Early 20th century	Solanaceae, Brassicaceae, Malvaceae, and Asteraceae	(Ishibashi et al., 2023)
Tomato mottle mosaic virus (ToMMV)	First reported in Mexico then in USA (California, Florida, New York, South Carolina), Brazil, Czech Republic, Spain, Israel, China, Iran, Australia	2013	Solanaceae, and Amaranthaceae	(Padmanabhan et al., 2025)
Tomato brown rugose fruit virus (ToBRFV)	First reported in Jordan and Israel, then in Germany, Italy, France, UK, Spain, Switzerland, Greece, Norway, Slovenia, Netherlands, Albania, Iran, Lebanon, Saudi Arabia, Syria, Turkey, Mexico, USA, Canada, China, Australia, Victoria	2015	Solanaceae, Amaranthaceae, Apocynaceae, and Asteraceae	(Carr, 2024; Chanda et al., 2021)
Cucumber green mottle mosaic virus (CGMMV)	First reported in England and later spread to Western Europe (Great Britain, Netherlands, Spain), Asia (Japan, India, Taiwan, South Korea), top East (Israel, Saudi Arabia), Eastern Europe, China, Southeast Asia, Southern Europe, North America, Australia	1935	Cucurbitaceae, Solanaceae, and Passifloraceae	(Ainsworth, 1935; Dombrovsky et al., 2017)
Pepper mild mottle virus (PMMoV)	First reported in Japan and then in Australia, China, Taiwan, North Africa, Europe, USA (Georgia, Florida)	Mid-20^th^ century	Solanaceae, Leguminosae, and Apocynaceae	(Secrist and Ali, 2018; Yu et al., 2018; Zamfir et al., 2023)

## 3 Economic impact and market losses

Some tobamoviruses species, such as ToBRFV and CGMMV, are reported to cause significant yield losses. Tobamoviruses infection can reach nearly 100% in greenhouses, resulting in a 30-70% production loss. This includes a reduced harvest period (fruit clusters per plant decrease from an average of 24–30 to 8-10) and unmarketable produce due to visual defects and deformities, making crops virtually useless (García-Estrada et al., 2022). The prevalence of CGMMV infections in cucurbit crops has routinely exceeded 25-40%, resulting in equivalent 20-40% losses in fruit yield and quality in melon and cucumber production regions that lack sanitation and resistant cultivars (Asad et al., 2022). The incidence of CGMMV on cucurbit farms in Punjab, Pakistan, was estimated at approximately 26-28%, with prevalence reported as high as 37%, suggesting that local cucurbit farms may suffer severe economic losses (Asad et al., 2022). In Mexico, ToBRFV reached all tomato-growing areas within less than a year since its detection in 2018. Despite limited data, qualitative reports indicate substantial market value loss and increased imports due to export reductions (García-Estrada et al., 2022). Mixed virus infections can cause greater damage than single-virus infections. For example, sweet potato plants co-infected with sweet potato feathery mottle virus (SPFMV) and sweet potato chlorotic stunt virus (SPCSV) suffer yield losses as high as 90% compared to much milder losses when each virus infects alone (Mukasa, 2004). Disease models and economic analyses indicate an insidious risk of vertical transmission from infected seeds. A low-frequency seed infection (~0.08%) can trigger outbreak, leading to global seed trade and regulatory embargoes. These interruptions incur indirect economic costs including seed testing, regulatory conformity, and loss of export business (García-Estrada et al., 2022).

## 4 Genome organization and replication

Tobamoviruses have helical symmetry with an optimum size of about 18 x 300nm^2^ with central core of 4nm in diameter. Its particle size contains 95% coat proteins and 5% nucleic acid and are composed of positive sense ssRNA whose genome is encoded in 6.4 kbp RNA molecule (Gomaa and Garcia-Ruiz, 2025). Tobamoviruses are composed of four open reading frames (ORF’s) with a 7-methylguanosine 5′ triphosphate cap at the 5′ terminus and three consecutive pseudoknots followed by a transfer RNA-like structure at the 3′ untranslated region (Dorokhov et al., 2018). ORF1 and ORF2 are present at 5′ end of viral genome and associated with replication associated proteins. ORF1 and ORF2 encodes for 126 kDa and 183 kDa proteins. These proteins contain enzymes like helicase, methyltransferase, RNA-dependent RNA polymerase (RdRp) etc. which further play their role in viral replication and transcription (Dorokhov et al., 2018). ORF3 encodes for movement proteins (MP) and is about 30 kDa in size (Dorokhov et al., 2018). It facilitates the virus particle to move from one cell to its adjacent cell through plasmodesmata. ORF4 is of 17.5 kDa and is located near 3′ end and encodes coat protein (Salem et al., 2016). CP plays a crucial role in encapsidation of virus particles to give its normal rod-shaped structure and is also responsible for host interaction and virus movement in some cases (Salem et al., 2016).

**Figure 1:**
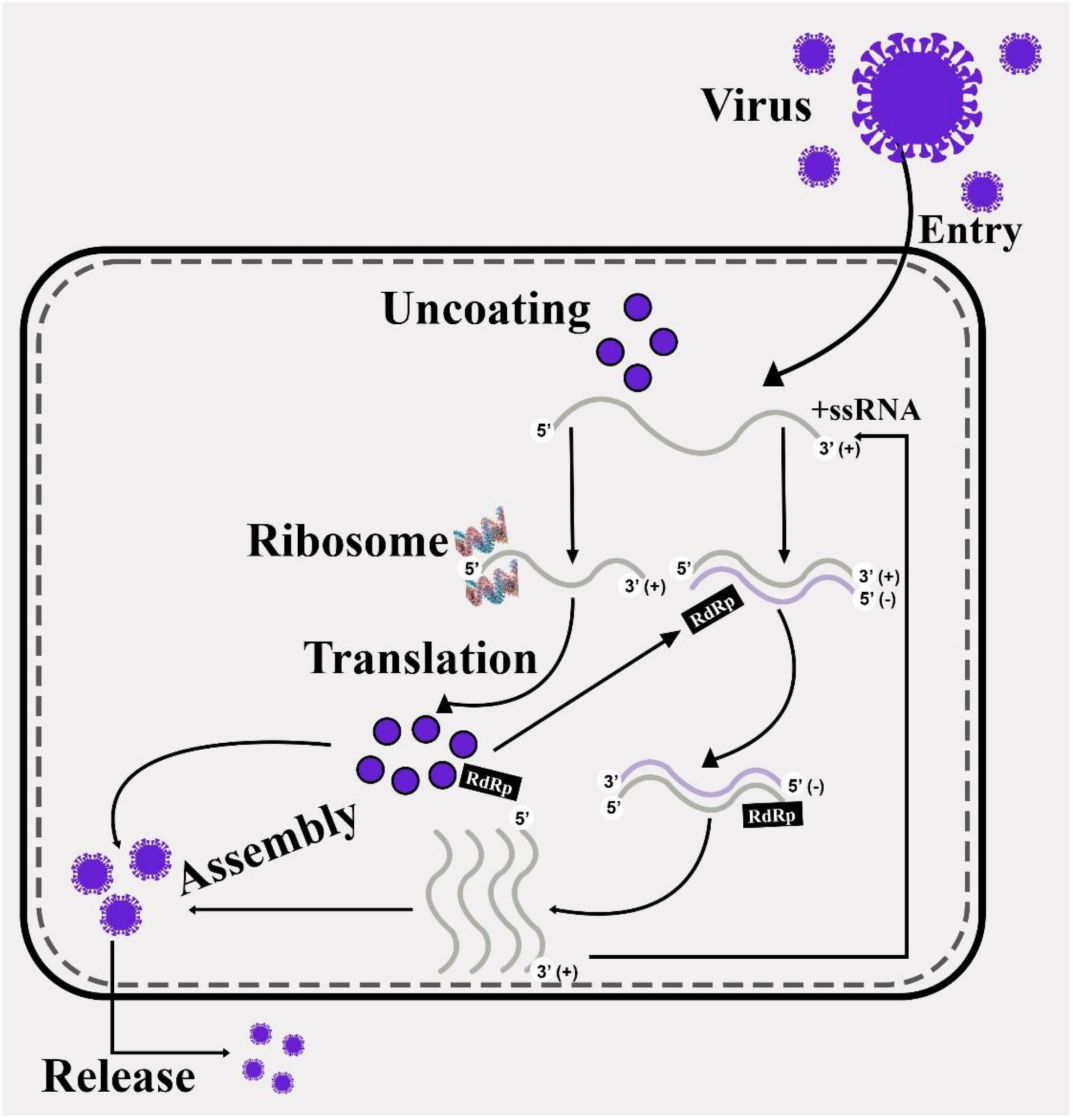
Replication of tobamoviruses (After entry, the virus uncoats itself, releasing positive-sense RNA. The genome serves as mRNA for the translation of replication proteins. These proteins synthesize complementary negative-strand RNA, which acts as a template for new positive strands. Genomic RNA and proteins assemble into virions and then released.).

Tobamovirus replication occurs in cytoplasm of host cells. Upon entry, the virus particle uncoates, exposing its RNA for replication. It hijacks the host cell’s protein synthesizing machinery (ribosomes) to initiate the replication of associated proteins, specifically, 126-kDa and 183-kDa proteins (Ershova et al., 2025). These proteins then form the replication complex, which synthesizes the complementary negative-strand RNA. This negative-strand serves as a template for producing new positive-strand genomic RNA. Transcription of subgenomic RNAs (sgRNAs) also occurs during replication. These sgRNAs, which are shorter than full-length genomic RNA, are responsible for producing movement protein (MP) and capsid protein (CP). The sgRNAs ensures the efficient production of proteins required at different stages of the virus life cycle (**Figure 1**) (Geng et al., 2021). Most viral genes perform their function inside the cell only in the presence of specific proteins, lipids or nucleic acids. Viral gene products directly contact these molecules and tobamoviruses follow the same mechanism. Their replication proteins not only participate in viral genome replication but also play a role in the host’s counter-defense mechanisms, such as against RNA silencing mechanism of host plants (Ishibashi et al., 2010).

Host factors involved in tobamovirus replication include two host-encoded proteins: TOM1 (a multi-pass transmembrane protein) and ARL8 (a small GTP-binding protein). These proteins are essential for the formation and activation of viral replication complexes on cellular membranes. The TOM1 protein associates with the viral helicase domain, anchoring replication proteins to membranes. Similarly, the ARL8 protein enables negative-strand RNA synthesis and 5′ capping of progeny RNA. Other host proteins recruited include TOM2A and TOM3, which maintain replication complex integrity, and translation-related proteins such as eEF1A. These proteins enhance overall efficiency of RNA synthesis (Yamanaka et al., 2000).

## 5 Transmission pathways

**Figure 2:**
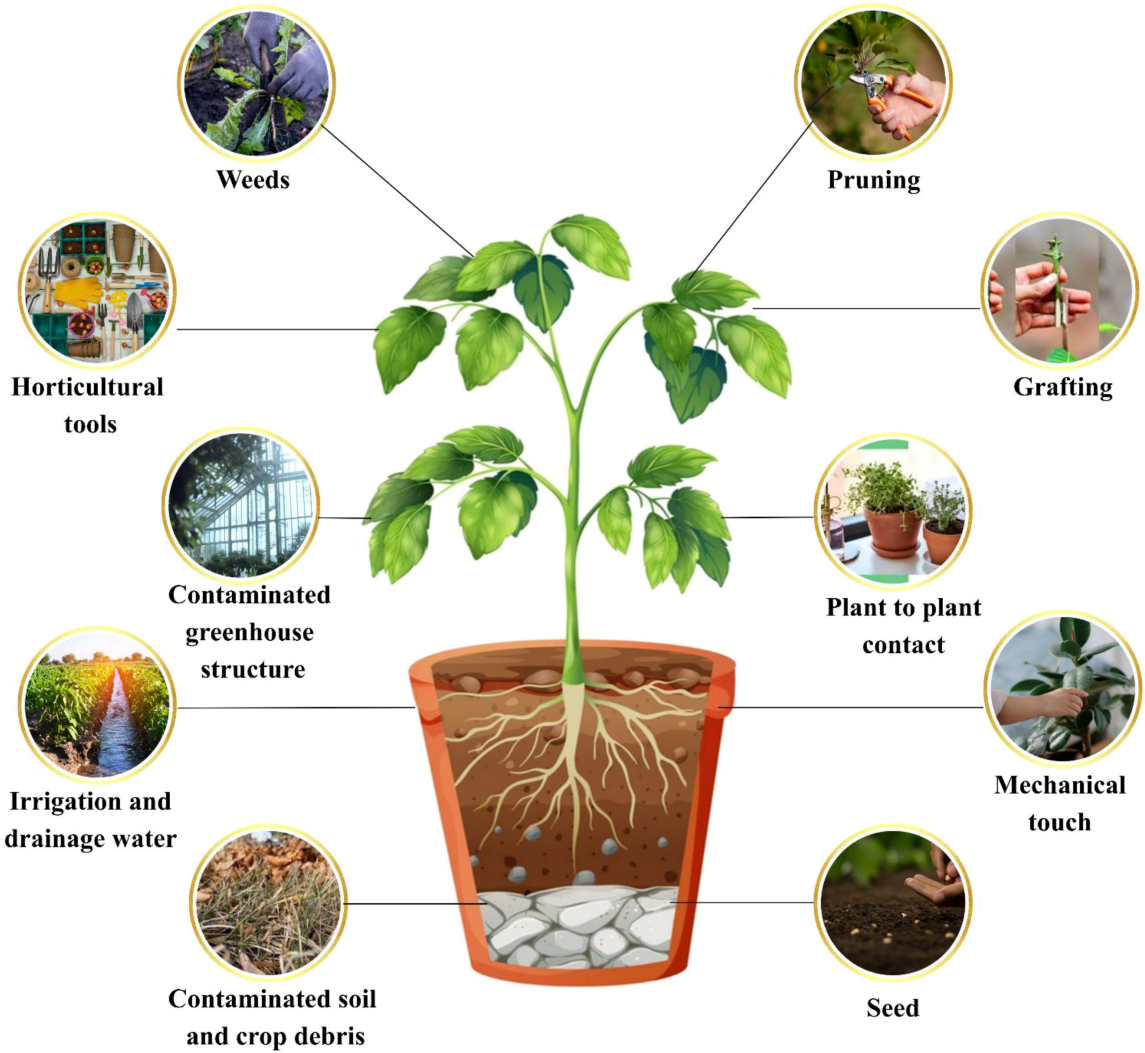
Transmission pathways of tobamoviruses depicting all possible means including mechanical means, contaminated seed, soil, and crop debris, irrigation water, plant to plant contact, grafting, pruning, weeds, contaminated greenhouse structures, and horticultural implements.

Tobamoviruses are a diverse group of plant pathogens known for their efficient mechanical transmission efficiency and environmental stability. They retain infectivity even after drying on hands, tools, greenhouse structures, plant debris, soil, or in water, which facilitates their spread during routine agricultural activities (Koh et al., 2018). Pruning, trellising, and transplanting infected plants further aids the movement of virions on tools, gloves or hands as mentioned in **Figure 2**. In CGMMV, 86% of its spread occurred within three weeks due to contact by workers, and pruning activities caused 11-32% infection based on 40 days of data (Smith and Dombrovsky, 2019). Similar patterns are well documented for TMV and ToBRFV, confirming mechanical contact as their principal route (Koh et al., 2018). Another important pathway is seed-mediated transmission. All seeds sampled from infected tomato fruits showed external virions on the seed coat; however, the infection rate of seedlings was only 0.08% (Salem et al., 2022). Watermelon seeds highly contaminated with CGMMV showed no direct seedling transmissions when grown, but induced disease when crude seed extracts were mechanically inoculated into healthy hosts (Sui et al., 2019).

Greenhouse management of tobamoviruses involve implementing strict hygiene, quarantine, and disinfection protocols to prevent mechanical transmission, cross-contamination, and rapid viral spread among crop plants. A comprehensive survey from 2019–2021 on tobamovirus contamination prevalence in tomato and capsicum seed lots revealed an 18-26% contamination prevalence, typically with very low prevalence rates (0.004-0.388%) (Dall et al., 2023). Even these low levels of inoculum can cause outbreaks in intensive agriculture areas due to their efficient mechanical dispersal. Bumblebees (B*ombus terrestris*), used in tomato pollination, can carry ToBRFV and TMV virions on their bodies and transmit them to adjacent plants in greenhouses (Avni et al., 2022). Honeybees (*Apis mellifera*), which graze on cucurbits infected with CGMMV, have also transmitted the virus to non-infected melons or cucumbers seedlings. While the virus does not internally infect pollen (and is not pollen-transmitted in ToBRFV), it adheres to pollinator bodies or pollen clumps and is mechanically transferred during buzz pollination (Avni et al., 2022). Environmental routes include soil and water-mediated transmission. TMV, CGMMV and yam mild mosaic virus (YTMMV) are some of those tobamoviruses that can infect plants through root uptake of contaminated irrigation water or through root-to-root infections, but the methods are less efficient. Although root-to-root transmission was uncommon for YTMMV, but viral particles could persist in soil and water for extended periods (Koh et al., 2018; Klein et al., 2023).

## 6 Virus-host interactions

**Figure 3:**
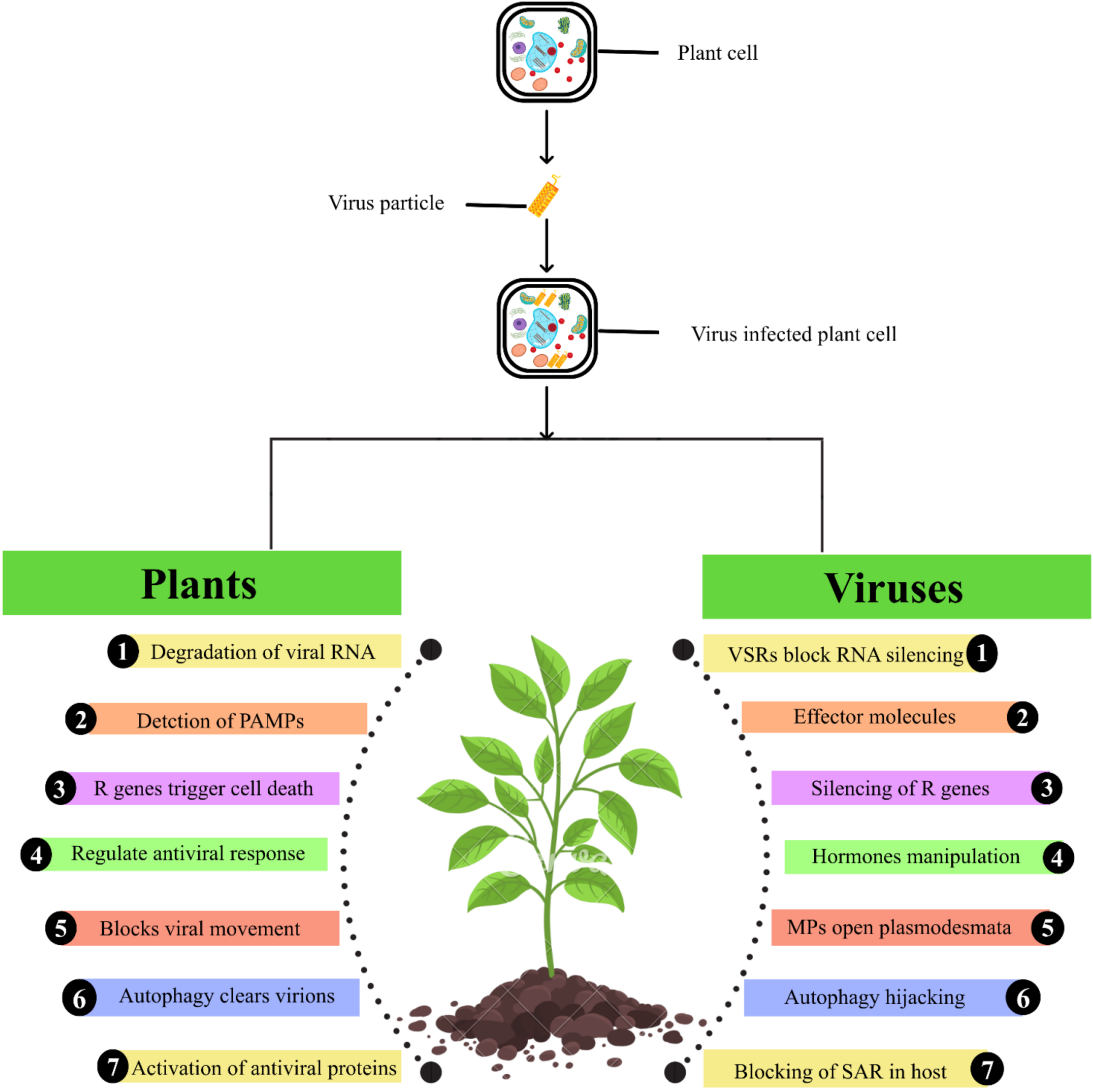
Plant defense mechanisms and viral counter strategies. Plants activate multiple immune responses, including RNA degradation, PAMP detection, R-gene activation, restriction of viral movement, autophagy, and antiviral protein production. In contrast, viruses utilize these responses through viral suppressors of RNA silencing (VSRs), effector proteins, R-gene silencing, hormonal manipulation, opening of plasmodesmata, autophagy hijacking and systemic acquired resistance (SAR) pathways to counter the plant antiviral defense.

Tobamoviruses interact with plant hosts through both structural proteins and by suppressing innate defenses, which shapes host specificity and disease outcomes and the same host plant uses a variety of mechanisms to suppress viral infection as mentioned in **Figure 3**. One of the crucial components of cell-to-cell transmission is movement protein (MP), as it contacts host plasmodesmata-associated proteins and the cytoskeleton (Wang, 2021). For example, MP associates with host actin, myosin, synaptotagmins, remorins, calreticulin, and pectin methylesterases. This association localizes MP to plasmodesmata, increasing their permeability and facilitating viral movement beyond innate immunity barriers (Ershova et al., 2023). MPs can inhibit pathogen triggered immunity (PTI) and subsequent deposition of callose at plasmodesmata which facilitates viral transmission (Kan and Citovsky, 2025). Additionally, CP and replicase proteins influence host processes. The coat protein aids in forming replication complexes and affects early mRNA translation of MP, thereby accelerating infection and preventing activation of host defenses (Dorokhov et al., 2017).

The replicase proteins of tobamoviruses (e.g. ~130 kDa or 125 kDa subunits) act as viral suppressors of RNA silencing (VSRs) by binding to small interfering RNAs (siRNAs). This disrupts key RNAi processes, such as HEN1-mediated methylation and Argonaute loading, thereby suppressing antiviral defense signals in plants and enabling more effective viral replication and spread (Zheng et al., 2024). In TMV and oilseed rape mosaic virus (ORMV), these suppressors inhibit systemic transmission of antiviral silencing, leading to complete infection (Sheshukova et al., 2020; Kan and Citovsky, 2025). The MP facilitated the cell-to-cell movement of TMV while also blocking PTI responses induced by dsRNA originating from plants (Ershova et al., 2025).

Transcriptional profiling of the host suggests a widespread immune response to TMV infection. Experiments using chimeric TMV strains in *Nicotiana tabacum* triggered strong upregulation of receptor-like kinases (e.g., FLS2, BAK1), defense regulators (EDS1, WRKY, NAC, MAPKKKs), and heat shock proteins. The phytohormone signaling pathways (SA, ABA, ET) were similarly modulated to mount pathogen-triggered and hormone-mediated immunity (Yu et al., 2020). Complex interactions arise in mixed infections. Plants also carry NB-LRR resistance genes such as *L* in pepper and *Tm-2²* in tomato. These genes recognize viral avirulence proteins to trigger effector-triggered immunity (ETI). The *L* gene detects the virus CP, while *Tm-2²* recognizes the MP of tobamoviruses. However, MP of ToBRFV harbors specific sequence changes that allow it to evade recognition by *Tm-2²* enabling the virus to infect resistant tomato varieties (Zhang et al., 2022). Finally, chloroplast interactions are critical. The RCR, psbO, and PSII proteins are chloroplast proteins linked with RCR, and psbO, thereby disrupting photosynthetic electron transport and leading to retrograde signaling. Both contribute to disease symptoms and regulate plant defense (Rahoutei et al., 1998).

## 7 Molecular surveillance tools for tobamovirus detection

**Figure 4:**
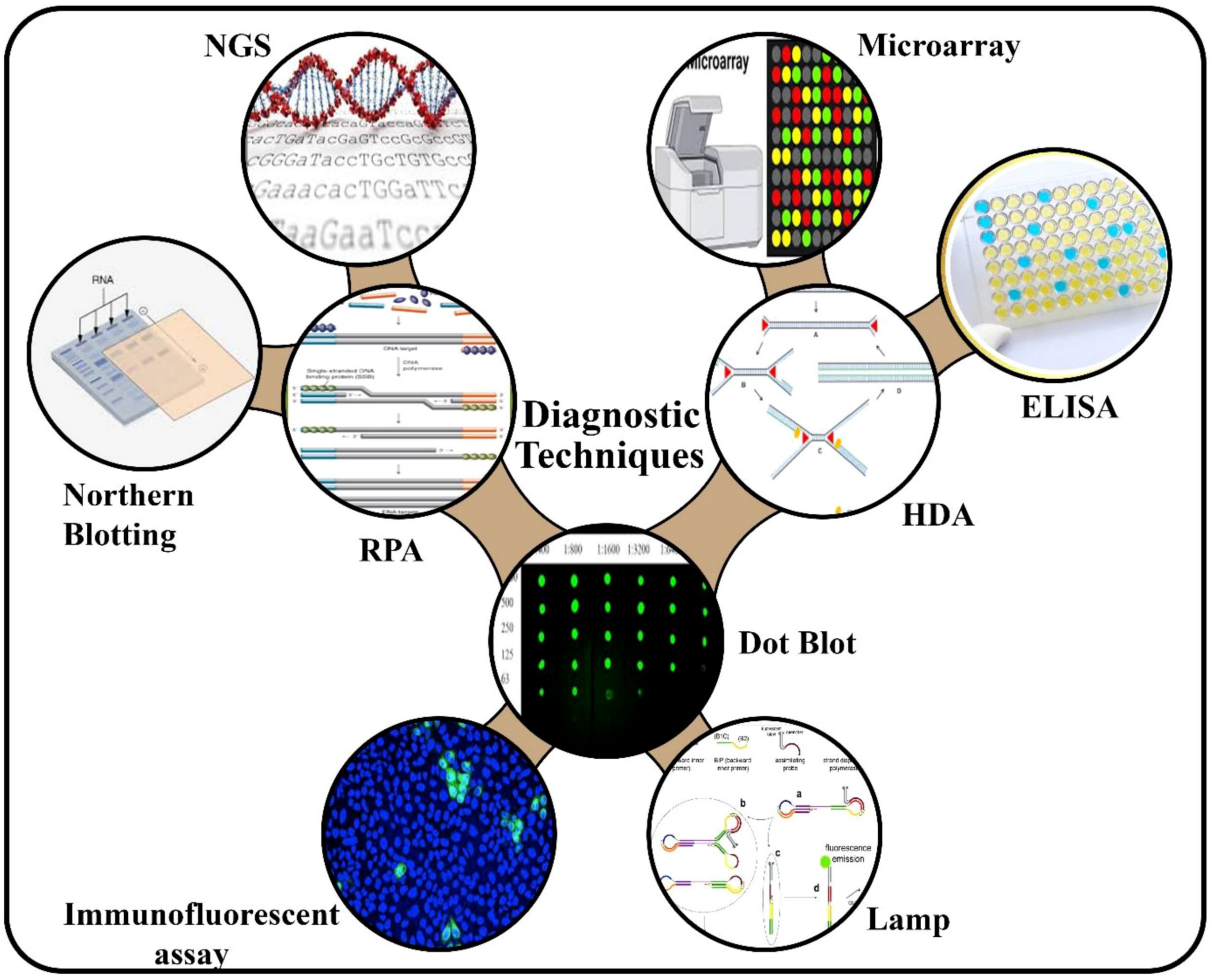
Advanced molecular tools for diagnosis of tobamoviruses (NGS, Next Generation Sequencing; RPA, Recombinase Polymerase Amplification; HDA, Helicase Dependent Amplification; ELISA, *Enzyme-Linked Immunosorbent Assay; LAMP,* Loop‐Mediated Isothermal Amplification).

Diagnostic methods have advanced significantly from traditional ELISA and RT-PCR, incorporating novel techniques like isothermal amplification, CRISPR/Cas systems, next-generation sequencing (NGS), and user-friendly field tests as mentioned in **Figure 4**. These innovations offer increased speed, accuracy, and ease-of-use. Reverse-transcription loop-mediated isothermal amplification (RT-LAMP) has shown great potential. For ToMMV, a new RT-LAMP using toothpick sampling eliminated RNA purification and achieved specificity against closely related tobamoviruses due to primers targeting the viral capsid protein. This technique demonstrated a ten-fold higher sensitivity than conventional RT-PCR, yielding results within 30 minutes (sensitivity ~10 folds to PCR). LAMP-based detection of ToBRFV has also been reported with high sensitivity and potential on-site application when lateral flow reagents are used as a readout (Zhang et al., 2022).

Recombinase polymerase amplification (RPA) can be integrated with CRISPR/Cas12a. A one-pot RT-RPA-CRISPR/Cas12a assay detects plant RNA viruses, such as tobamoviruses, within 30 minutes at a single temperature, with results visualized by fluorescence making it ideal for field diagnostics (Aman et al., 2020). The sensitivity of the LbCas12a, RT-LAMP, and lateral flow strips for point-of-care detection of ToBRFV within 25 minutes was equivalent to that of RT-qPCR (Bernabé-Orts et al., 2022). Distinguishing closely related species in *Tobamovirus* genus has been exceptionally complicated; however, CRISPR has simplified this process. The CRISPR/Cas12a systems can differentiate ToBRFV and ToMV in field samples. Their sensitivity is comparable to lab-based RT-PCR, capable of detecting single-copy targets. Furthermore, they can be performed using portable devices, such as hand warmers, for heating (Alon et al., 2021). CRISPR/Cas13a has been developed for extraction free, mobile-phone based detection of ToBRFV in tomato leaves, distinguishing it from other related viruses even at asymptomatic or early stages of infection within ~15 minutes (Hak et al., 2024). CRISPR/Cas9 genome editing has become a powerful tool for investigating host-virus relationships and inducing resistance (Jogam et al., 2023).

New advanced sequencing technologies, such as high-throughput and portable sequencing, improve seed lot screening. Compared to conventional ELISA or PCR. Oxford Nanopore sequencing identified ToBRFV-infected seeds with higher sensitivity, detecting a single infected seed among 200 uninfected ones (Smith and Dombrovsky, 2019). Next generation sequencing (NGS) also clarifies viral genetic diversity, facilitates lineage tracking, and aids in developing species-specific assays. Multiplex RT-qPCR techniques can simultaneously detect multiple viruses, such as ToBRFV, TMV, ToMV, TSWV in a single reaction thereby increasing throughput and economizing testing costs (Zhang et al., 2022). An RT-PCR duplex real-time protocol for ToMMV and ToBRFV, developed according to EPPO guidelines, has demonstrated effective sensitivity, specificity and applicability for seed and leaf diagnostic tests (Tiberini et al., 2022). Serological methods for detecting ToBRFV have improved in accuracy by using monoclonal antibodies that target unique CP epitopes. These antibodies are used in ELISA and colloidal-gold immunochromatographic strips tests. The strips yields specific results in just five minutes and do not react with other similar viruses, make them highly reliable (Ghorbani et al., 2024; Zhao et al., 2024).

Universal degenerate-primer RT-PCR assays are also important for broad-range detection. Li et al. (2018) designed a primer pair based on the sequences of conserved replicase genes that produce amplicons in at least nine tobamovirus species (including TMV, ToMV, ToMMV, PMMoV, and CGMMV) and can be used cost-effectively in mixed infections. Wastewater samples from Southern California were sequenced to study tobamoviruses over the course of a year. Scientists identified thousands of single nucleotide variants (SNVs) across eight distinct tobamovirus species. This method revealed the evolutionary pattern of viruses and their changing prevalence across the community (Rothman and Whiteson, 2022). Bioinformatics and machine learning approaches combined with long-read data offer future potential for *de-novo* assembly and detection of novel viral haplotypes or structural variations (Shim, 2019).

Transcriptomic and genomic data provide useful information about the host’s reaction to tobamovirus infection. Transcriptome sequencing in tomatoes inoculated with ToBRFV identified 522 differentially expressed genes (DEGs), many of which belonged to wound response, protein processing, response to stress and defense signaling pathways. A total of ten DEGs were verified by qRT-PCR, indicating their potential as analysis markers or candidate resistance-related genes (Thakare et al., 2024; Wang et al., 2024a). A complementary ionomics-transcriptomics study of resistant tomatoes found significantly higher leaf iron and nickel concentrations. The expression of genes related to iron homeostasis, K+ transporters, LRR signal receptors, and chitinases was also increased, pointing to combined nutrient and transcript patterns as an early screening tool for resistance (Thakare et al., 2024; Canto-Pastor et al., 2019)(**Table 2**).

**Table 2:** Multi-Omics approaches for improved diagnosis and management of tobamoviruses.

Omic approach	Crop	Target tobamovirus	Application	Reference
Transcriptomics	Tomato	Tomato brown rugose fruit virus (ToBRFV)	Complete genome characterization, strain differentiation	(Thakare et al., 2024)
Proteomics	Tobacco	Tobacco mosaic virus (TMV)	Revealed proteins involved in viral replication and movement	(Di Carli et al., 2012; Das et al., 2019)
Metabolomics	Tomato	TMV	Identified altered primary metabolites and defense-related compounds	(Li et al., 2025)
Phenomics	Tobacco	TMV	Early detection of symptom expressions using imaging platforms	(Shashko et al., 2021)
Epigenomics	Cucumber	*Cucumber green mottle mosaic virus* (CGMMV)	Showed altered epigenetic regulation of defense genes	(Sun et al., 2019)
Small RNAomics	Tomato	*Tomato mosaic virus* (ToMV)	Revealed vsiRNA-mediated host antiviral silencing	(Turco et al., 2018)
Lipidomics	Tobacco	Different tobamovirus species	Identified lipid-mediated regulation of viral replication sites	(Samarskaya et al., 2025)
Phosphoproteomics	Tobacco	TMV	Identification of phosphorylation changes in host proteins upon virus infection	(Lu et al., 2019)
Population genomics	Tobacco	TMV	SNP/phylogenetic analyses across isolates (Nextstrain)	(McLeish et al., 2021)

Proteomics studies have enabled the direct detection of viral and host response protein markers. Tomato nanovesicles were analyzed using mass spectrometry, and the ToBRFV coat protein was detected with approximately 55% peptide coverage. This suggests these vesicles could serve as a sensitive method for detecting the virus before symptoms development. In tobacco infected with TMV, the iTRAQ proteomics identified more than 400 differentially abundant proteins related to photosynthesis, oxidative stress, protein processing, and defense signaling. This indicated systemic host responses involved in biomarker discovery (Das et al., 2019; Mammadova et al., 2021). Metabolomics studies in tobamovirus research are still emerging. Recently, elevated levels of osmolytes, including proline, myo-inositol, and trigonelline, have been reported in infected tissues. These compounds relate to viral load and plant stress adaptation, alluding to their viability as non-invasive plant metabolic identifiers of early viral infection in tomato and cucumber (Salmerón et al., 2025). Recently, small RNA sequencing in tomato plants infected with ToBRFV has shown a large abundance of 21 and 22 nucleotide vsiRNAs. Most of these vsiRNAs mapped hotspots in the RdRP, movement protein, and coat protein. with many exhibiting a 5′nucleotide bias (uridine-rich). RT-PCR validation confirmed their accumulation. Predicted vsiRNAs host gene targets were significantly downregulated. Therefore, vsiRNAs are not only antiviral factors but also dictate host gene expression. This can be used to develop highly specific molecular signatures to diagnose an infection and potentially develop RNAi derived resistance mechanisms (Chen et al., 2025).

The classical R genes *Tm-1* and *Tm-2²*, originally identified in wild tomato species (*Solanum habrochaites* and *S. peruvianum*), confers strong resistance against TMV and ToMV when introgressed into *S. lycopersicum*. However, these genes show weak or no efficiency against ToBRFV, which has developed movement proteins variants that evade recognition by *Tm-2²* (Jewehan et al., 2022; Zinger et al., 2025). *Tm-1* codes for a protein that interacts with viral replicase, thereby preventing ToMV replication. In contrast, *Tm-2²* encodes a CC-NBS-LRR receptor that interacts with viral movement protein, inducing the effector-activated immunity (van Damme et al., 2023). Recent studies have identified new genetic sources of novel tolerance and resistance in wild tomato accessions. Zinger et al. (2025) discovered that the combination of dominant resistance locus *Tm-1* with a novel dominant locus in chromosome 11 provides effective resistance to ToBRFV in tolerant tomato plants. The overexpression of *Tm-1* in a tolerant genotype significantly reduced viral accumulation, while silencing the *Tm-1* locus converted susceptible genotypes into tolerant ones. This demonstrates a synergetic interaction between recessive and dominant loci.

Jewehan et al. (2022) screened wild tomato species (*S. habrochaites* and *S. peruvianum*) and identified several accessions with strong resistance to ToBRFV at approximately 24 °C. However, this resistance failed at higher temperatures due to a mutant viral isolate, Tom2M-Jo. This mutant carries two mutations in the movement protein. These mutations (Phe22→Asn and Tyr82→Lys), allow the virus to overcome innate immunity conferred by wild species. This highlights how single amino acid changes in MP can break temperature-sensitive plant defenses (Sánchez-Sánchez et al., 2022). Moreover, a variety of environmental factors including high temperatures can break or weaken resistance responses. For instance, wild tomato accessions with latent resistance to ToBRFV, showed symptoms at 33 °C, but not at 24 °C, suggesting that abiotic stress can promote resistance breakdown (Sánchez-Sánchez et al., 2022). Low genetic diversity among commercial cultivars of tomatoes also accelerates erosion of resistance because similar host genetics exerts very strong selection pressure on the virus and permits adapted strains to develop rapidly (Gomaa and Garcia-Ruiz, 2025). In pepper (*Capsicum annuum*), resistance gene alleles L¹–L^4^ offer protection against PMMoV, but the plant is only transiently tolerant to systemic infection when infected by ToBRFV, frequently followed by hypersensitive leaves. Nevertheless, fruits and their seeds are asymptomatic and non-infectious, indicating partial or tolerance-based resistance rather than complete immunity (Eldan et al., 2022). Other ongoing studies are pursuing bioinformatic-directed mutagenesis in the *Tm-2^2^* receptor to restore and expand recognition of the ToBRFV movement protein variations. Rivera-Márquez et al. (2022) established that resistance could be obtained through precise CC-NBS-LRR, leading to resistant transgenic tomato lines. Future efforts will likely focus on high priority stacked or polygenic breeding types of resistance. This will involve classical R genes, novel tolerance loci, and knockouts of susceptibility genes using genome editing to generate durable and broad-based protection.

In addition to the MP, the replicase region (p126) is also under selective pressure. Sequencing of Canadian isolates from greenhouses and wastewater has shown non-synonymous polymorphisms in the replicase region (p126). These mutations can decrease the affinity of Tm-1 or other resistance gene products for the virus. This indicates that resistance-breaking mutations do not exclusively occur in the MP but can also be found in the replicase, enabling evasion of host factor recognition (Di Rosa et al., 2025; Fougere et al., 2025). Resistance to tobraviruses is also temperature sensitive. Both wild accessions, *S. habrochaites* and *S. peruvianum*, become susceptible following infection at 33 °C, whereas normal resistance levels were observed at 24 °C (Mushegian et al., 1989). Additionally, ToBRFV-Tom2M-Jo mutant can overcome this innate immunity at any temperature, demonstrating its capacity to rapidly induce resistance breakdown through both environmental and mutational mechanisms (Jewehan, 2022). These studies point out various mechanisms by which tobamoviruses overcome host resistance, including mutations in the MP protein that interfere with EDRF receptors recognition, attenuated viral movement proteins, replicase polymorphism that evades Tm-1 induced inhibitory binding, and environmental influences on host resistance. Evidence shows that resistance breakdown arises from a delicate balance between viral virulence and immune activation, where minor changes in viral proteins enable escape with modest fitness costs. These insights highlight the need to have long-term resistance mechanisms, such as gene-stacking with multiple R genes, targeting both replicase and recognition, and combining recessive gene edits to susceptibility genes to reduce the possibility of breakdown.

## 8 Management innovations for tobamoviruses

### 8.1 Disruption of host susceptibility genes

Host plants can be engineered to manage viral diseases by manipulating their virus susceptibility genes. Gene editing technologies, such as CRISPR-Cas9, provide target-specific gene disruption in host plants. Tobamoviruses require several host proteins for replication in tomato plants including TOM1 (Yamanaka et al., 2000), TOM2A (Tsujimoto, 2003), which are multi-pass transmembrane proteins. Additionally, GTP-binding proteins like ARL8 also perform this function (Nishikiori et al., 2011). In *Arabidopsis thaliana, AtTOM1* and its two homologs, *AtTHH1* and *AtTOM3*, along with *AtARL8a, AtARL8b*, and *AtARL8c* are three homologous genes that encode ARL8. Knockouts of *AtTOM1* and *AtTOM3* confer resistance against tobamoviruses, including ToMV, without affecting the growth and yield of tomato plants (Fujisaki et al., 2006). Similarly, knockout of *AtARL8a* and *AtARL8b*, also confer resistance to tobamoviruses in tomato plants (Nishikiori et al., 2011). A knockout of *TOM2A* in *A. thaliana* develops resistance to ToMV, but it has little or no effect on its multiplication. In both cases, the engineered plants remain susceptible to CMV. ARL8 and TOM1 proteins interact both with tobamovirus replication and with each other. These interactions regulate the enzymatic activity of the tobamovirus replication protein. Consequently, the viral negative strand of RNA cannot be synthesized in the absence of these host factors (Nishikiori et al., 2011).

Hu et al. (2021) recently engineered tomato plants by knocking out *AtTOM2A*, resulting in resistance to ToMV and TMV. Similarly, Kravchik et al. (2022) used CRISPR-Cas9 technology to knock out susceptible genes, including *SlTOM1a, SlTOM1b*, and *SlTOM1c* in the tomato cultivar ‘M82’. They developed *SlTOM1c* double and *SlTOM1abc* triple mutant tomatoes. The study concluded that the accumulation of ToBRFV coat protein (CP) was slower in both mutants compared to wild cultivars. The triple mutant plants also showed delayed accumulation of ToMV CP. Ishikawa et al. (2022) utilized the same technology to knockout *SlTOM1a–d* gene in the cultivar ‘GCR26’. The study resulted in mutant lines containing single or multiple *SlTOM1* mutations. In single and double mutants, the accumulation of ToBRFV and ToMV coat protein was similar or slightly lower than that in wild non-transgenic type. Triple mutant plants showed slower CP accumulation for both ToBRFV and ToMV compared to the wild type. No CP accumulation of ToBRFV and ToMV, nor any disease symptoms, were observed in quadruple-mutant plants. These findings suggest that four *SlTOM1* genes are involved in the multiplication of ToBRFV and ToMV in parallel following the order of functional importance: *SlTOM1b* < *SlTOM1d* < *SlTOM1c* ≤ *SlTOM1*a. *SlTOM1* triple mutant plants showed strongest resistance when inoculated with ToBRFV, and the virus was unable to replicate in *Sltom1* quadruple mutant tomato plants. No tobamovirus mutant has yet emerged with the ability to multiply in *Sltom1* quadruple mutant plants. These results are promising, although the durability of resistance still requires field evaluation. Additionally, researchers are studying recessive resistance mechanisms involving plant genes essential for viral replication, such as TOM1, TOM2, and TOM3-like genes. These are not traditional R genes but rather susceptibility genes (S-genes), as viruses rely on them for replication within the plant. Functional studies and CRISPR/Cas9 knockout experiments in tomato and Arabidopsis have shown that disabling TOM1 (and often TOM3) can completely suppress tobamovirus multiplication, including ToBRFV and TMV, without harming plant growth. These host factors are essential for tobamovirus replication, as demonstrated in knockout studies and could confer broad-spectrum resistance (Ishikawa et al., 2022) (**Table 3**). Such multiple approaches are inevitable due to ToBRFV breaking conventional resistance genes.

**Table 3:** Key resistance Genes/Alleles contributing resistance to tobamoviruses along with their targeted hosts.

Gene/allele	Host plant	Mode of resistance	Target virus	Reference
Tm-1	Cultivated tomato (*S. lycopersicum*)	Overexpression reduces viral accumulation; interacts with Chr 11 tolerance	ToBRFV	(Zinger et al., 2025)
Tm-2		CC-NLR; long-used tobamovirus R gene	TMV/ToMV (broken by ToBRFV)	(Hak and Spiegelman, 2021)
Tm-2² (Tm-22)		Durable for ~60 yrs; overcome by ToBRFV MP variants	TMV/ToMV	(Hak and Spiegelman, 2021; van der Gaag and Mehle, 2022)
Tm-1 + Chr 11 locus	Wild tomato (*S. habrochaites*)	Polygenic dominant resistance	ToBRFV	(van Damme et al., 2023)
L gene-mediated resistance	Pepper (*Capsicum annuum*)	Protects against newly identified tobamovirus species	New tobamovirus species	(Vélez-Olmedo et al., 2021)
L1a			Tobamoviruses	(Sawada et al., 2004)
L^4^ allele (temperature-sensitive)		Resistance compromised at high temperatures		(Eldan et al., 2022)
N′ resistance gene	Pepper and Tobacco	Typical R gene conferring resistance		(Scholthof, 2017)
WPRb	Watermelon (*Citrullus lanatus*)	Host factor tied to plasmodesmata influencing replication	CGMMV	(Cai et al., 2023)
SlTOM1 and SlTOM3 (S-gene)	Cultivated tomato (*S. lycopersicum*)	Knockout confers resistance	ToBRFV	(Kravchik et al., 2022)
Quadruple TOM1 KO (S-genes)		CRISPR knockout of four TOM1 homologs	ToBRFV/ToMV	(Ishikawa et al., 2022)
ARL8a/ARL8b (S-genes)	*Arabidopsis thaliana* (model for concept)	Small GTPases; required for tobamovirus replication with TOM1	Tobamoviruses	(Nishikiori et al., 2011)

Pathogen-derived resistance (PDR) is an essential strategy for managing tobamoviruses, relying on the use of viral genes, or derivatives, to confer protection in transgenic plants. The original and most well-studied approaches include coat-protein (CP)-mediated resistance, in which viral CP is expressed constitutively in a plant to reduce virus infection and symptom development (e.g., TMV/CP) (Beachy, 1999). The RNA-based form of PDR has evolved into RNAi, express hairpin or antisense constructs that will trigger sequence-specific degradation of viral RNAs; RNAi has provided durable resistance against several tobamoviruses in crop systems (Zhao et al., 2020). To a limited extent, replicase (RdRp) popularized PDR by using RdRp-derived constructs that could interfere with virus replication/assembly (Arif and Hassan, 2000). Regardless, transgenic PDR continues to suggest proven efficacy historically (TMV, ToMV), but transgenics for emerging strains (e.g., ToBRFV) to overcome classical R-gene utilization express the limitation of single-gene resistance, suggesting the value of exactly-stacked, or at least designs aiming for a multi-mechanism mentality (Carr, 2024). As a result, modern implementations of PDR will stress combinatorial designs (RNAi plus edited host susceptibility genes, or CRISPR-based immunity platforms) while focusing on molecular surveillance to understand the potential for viral escape (Gottula and Fuchs, 2009). Despite regulatory and public-acceptance issues restricting field application in some geographies, PDR has potential to be a valuable component of integrated tobamovirus management in combination with resistance-breeding, hygiene protocols, and viral surveillance diagnostics (Beachy et al., 1990).

### 8.2 Attenuated strains

When a mild strain of virus infects a plant, it can prevent or delay infection by another strain of the same or a closely related strain of same virus. The process is also called cross protection. Studies proposed two mechanisms by which cross-protection is achieved. In first mechanism, the coat protein of the mild strain of virus interferes with uncoating or replication of virus. In a second mechanism, RNA-silencing pathways are induced, in which there is change in the population of siRNAs that target both mild and the severe strain of that virus (Nishiguchi and Kobayashi, 2011). In the case of tobamoviruses, research has shown that the protective virus strains have mutations in the replicase or the suppressor region such that the mild strain loses pathogenicity, but retains the ability to replicate and to trigger RNA silencing that confers resistance to the virulent strain of tobamovirus (Carr, 2024). A variety of attenuated strains of tobamoviruses have been created and tested to employ in cross-protection of a given crop and reducing the severity of symptoms in plants. For example, UV and nitrous acid mutagenesis were used to generate the attenuated CGMMV-SH33b strain of CGMMV. This strain has successfully protected melon (*Cucumis melo*) crops by reducing viral load and symptoms. Mutation analysis showed that the attenuated phenotype and cross-protection were associated with changes in its replicase and MP genes (Ali et al., 2016). Similarly, PMMoV-C1421, a non-virulent variant of pepper mild mottle virus, was applied to *Capsicum annuum* to provide resistance against more aggressive strains. This strain demonstrated good prospects of non-toxic viral control in pepper crops on a commercial basis (Ogai et al., 2013).

Xu et al. (2024) engineered a double-mutant strain of TMV (TMV-R88A/S114R) to target two amino acids residues within the viral replicase p126. The first mutation changing the conserved arginine (R) at position 88 to alanine (A), which eliminated cell-to-cell movement and significantly reduced replication. The second mutation replaced serine 114 with arginine (S114R). The derivative TMV-R88A/S114R showed no apparent symptoms even after 70 days post-inoculation. The engineered mutations persisted and demonstrated genetic stability after 4 serial passages. When TMV-R88A/S114R pre-inoculated plants (14-day patency interval) were challenged with the wild-type TMV, no symptoms appeared, and a 68% reduction in coat protein was observed compared to non-protected plants. The efficacy of the protective strain in safeguarding these plants against the wild-type virus was confirmed by sequence analysis. The protective strain showed 100 percent chase-out of the wild-type virus, establishing its effectiveness as a cross-protective agent and a stable attenuated strain in disease management (Xu et al., 2024).

Slavokhotova et al. (2016) engineered begonia viruses, specifically CGMMV, by introducing single amino acid replacements in the coat protein and RdRp. These mutations lowered the virus considerably but not their capacity to produce systemic infections. The attenuated strains provided good cross-protection against severe CGMMV infection in cucurbits. When high concentrations of these mild mutants were inoculated into plants in advance, the plants did not show severe symptoms and experienced less accumulation of the wild-type CGMMV. This work identifies a promising molecular target that could induce the modification of stable protective CGMMV strains through mutagenesis. These examples emphasize the continued advancement of attenuated tobamovirus strains for disease management. Their application is especially pertinent as some strains have developed resistance to interventions, necessitating more sustainable solutions. Further fine-tuning of attenuated strains and their use in breeding and integrated management also show promise for research and implementation.

### 8.3 CRISPR and RNAi technologies

Both CRISPR and RNA interference (RNAi) technologies have potential durable resistance applications for tobamoviruses. They target either the host susceptibility genes or the viral genome itself. RNAi with dsRNA or hairpin RNAs targeting either the host or the viral genes has been reported to reduce levels of tobamoviruses (e.g., Tomato mosaic virus ToMV) in transgenic plants through the generation of specific siRNAs and lowered virus levels in subsequent generations. RNAi is a powerful antiviral method against viral genes. Tomato orthologs of TOM1 were knocked down using RNA silencing, which tremendously suppressed ToBRFV accumulation and improved virus resistance (Shahriari et al., 2023) Similarly, targeting the replicase subunit p122 of TMV and Arabidopsis-infecting tobamoviruses with RNAi was also effective in blocking the systemic spread of the virus by evading the virus suppressors of RNA silencing (VSRs) (Zheng et al., 2024).

**Figure 5:**
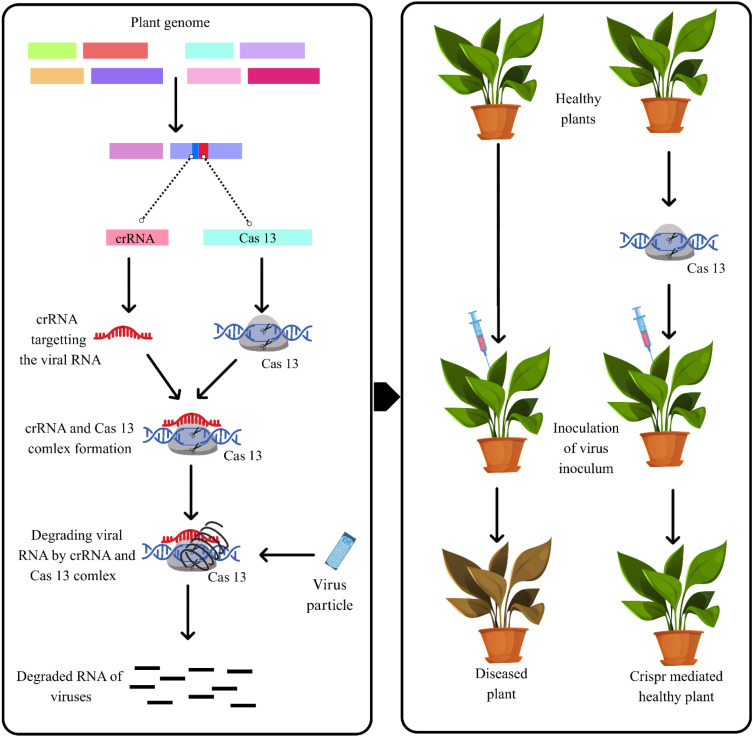
Mechanism of CRISPR in managing tobamoviruses (CRISPR RNA (crRNA) is designed to specifically target viral RNA sequence. The Cas13 enzyme, guided by the crRNA, forms crRNA-Cas13 complex that binds to the viral RNA. Cas13 cleaves and degrades the viral RNA after binding. It effectively neutralizes the virus, preventing its replication within the plant cell).

The CRISPR–Cas13 system provides antiviral defense targeting RNA by specifically degrading the viral RNA genome following infection of plant cells. To use the CRISPR–Cas13 system, a CRISPR RNA (crRNA) is designed to complement the conserved portion of the viral RNA genome; the crRNA then guides the Cas13 effector protein to the target sequence. Once the target has been bound by Cas13 and crRNA, the nuclease domains called HEPNs (Higher Eukaryotes and Prokaryotes Nucleotide-binding) are activated, which cleave and degrade the viral RNA, halting replication and movement of the virus in the plant. Data has shown that variants of the Cas13 protein, specifically Cas13a and Cas13d, can repress the replication and movement of the TMV and turnip mosaic virus (TuMV), reducing both viral load and symptom expression in infected plants. This RNA targeting provides a specific, programmable, and non-transgenic method to manage tobamoviruses as shown in **Figure 5**. A 2018 study in *A. thaliana* demonstrated its effectiveness, using LshCas13a with crRNAs targeting TuMV helper component protease (HC-Pro) and GFP regions to reduce viral accumulation by up to 50% (Aman et al., 2018). CRISPR-based assays are increasingly practical for plant virus detection. A 2024 review highlights that CRISPR/Cas12a and Cas13a/d systems can identify viruses within 30 minutes, producing visible results, on lateral flow strips or via fluorescence. No extensive RNA purification is needed, making these tests useful for monitoring tobamovirus infections directly in the field (Jaybhaye et al., 2024). All research demonstrates the evolving use of CRISPR and RNAi technology for tobamovirus management.

### 8.4 Bio-formulations

Biological agents, which include polysaccharides, peptides and small proteins, and beneficial microorganisms, have reliably elicited or enhanced plant resistance against tobamoviruses, in part by priming innate immune pathways, altering host metabolism, and sometimes directly interfering with the virus (Trojak-Goluch, 2024). For instance, plant growth-promoting rhizobacteria (PGPR) can induce systemic resistance (ISR) through the jasmonic acid and ethylene signaling pathways. They can also establish systemic acquired resistance (SAR) via the accumulation of salicylic acid and the expression of pathogenesis-related proteins and enzymes with antiviral activity. Additionally, some bio-agents are known to produce ribonucleases and other compounds that degrade viral RNA or inactivate coat proteins (Manjunatha et al., 2022). The polysaccharide elicitors (for example, chitosan; chitosan-oligosaccharides (COS)) have been one of the better documented examples; foliar applications and seed treatments using chitosan or COS were effective at reducing TMV and related tobamovirus accumulation when they induced reactive oxygen species (ROS), callose deposition, upregulated pathogenesis-related (PR) genes, and salicylic-acid-dependent defences; both meta-analyses and experimental papers have demonstrated prophylactic and curative effects based on molecular weight and dose (Komarova et al., 2024). Additionally, chitosan-based composites, when combined with alternative biological combinations i.e. cytosinpeptidemycin + COS, have further demonstrated synergistic antiviral impact, suggesting formulation chemistry could matter for final efficacy in a field setting (Guo et al., 2020). The polysaccharide obtained from the dry mycelium of *Penicillium chrysogenum* has ability to significantly induce resistance against TMV in tobacco plants. These polysaccharide act as an elicitor and enhance systemic resistance by activating defense-related enzymes (Fu et al., 2020).

Small proteins and peptides function as direct antivirals or powerful immune stimulants. Harpin proteins, when applied exogenously, reliably induce systemic acquired resistance (SAR)-like responses and were shown to reduce TMV symptoms and viral loads in host plants through activation of Mitogen-activated protein kinase (MAPK) cascades and expression of defense genes (Wang et al., 2020). Antimicrobial peptides (AMPs), such as thanatin or chimeric lactoferricin-derived peptides, have shown to have direct antiviral activity against TMV using half-leaf and leaf-disk experiments. Broader assessments of plant-derived AMPs indicate promise as templates for the design of antiviral agents targeting viral particles or host defense modulation (Sabokkhiz et al., 2019). Additionally, Zhong et al. (2021a) showed that the extracts obtained from *P. chrysogenum* restricted TMV spread in *N. benthamiana* by priming plant defenses, particularly enhancing callose deposition at plasmodesmata. The narrowing of plasmodesmata blocked the viral cell-to-cell movement (Zhong et al., 2015). In the same way *P. chrysogenum* polypeptide extract protected tobacco from TMV infection by regulating abscisic acid (ABA) biosynthesis and enhancing callose priming (Li et al., 2021).

Beneficial microorganisms, including rhizobacteria (*Bacillus*, *Pseudomonas*), fungal biocontrol agents (*Trichoderma* spp.), and endophytes, typically act indirectly by inducing systemic resistance or altering host physiology to minimize viral replication. Multiple investigations demonstrate that foliar or root applications of concentrated *Bacillus subtilis* cultures or viable inocula reduce TMV, ToMV symptoms and viral replication while enhancing antioxidant enzyme activities, phenolic content, and PR gene expression in the treated plant (El-Gendi et al., 2022). It is also reported that *Trichoderma* strains colonize in roots and stimulate systemic immunity in the host that reduces viral replication and disease severity in tomato and tobacco models, by their general ability to prime defenses, generate elicitor molecules, and increase overall vitality (Abdelkhalek et al., 2022). Moreover, another study demonstrated that crude peptides extracted from the dry mycelium of *P. chrysogenum* act as microbe-associated molecular patterns (MAMPs), triggering systemic resistance in tobacco and effectively reducing TMV infection (Zhong et al., 2021b). Earlier study indicate the mechanisms used by microbial protection against viruses including root-mediated ISR, a range of competitive interactions for niche and resource utilization in the phyllosphere or rhizosphere, the production of antiviral metabolites, and indirect activation of plant RNA-silencing pathways (Manjunatha et al., 2022).

From a mechanistic perspective, these biological agents often converge a few host responses, including: (1) early signalling (fluxes of calcium, MAPK activation), (2) oxidative burst and fortification of the cell wall, (3) activation of SA/JA/ET pathways and PR genes, and (4) enhancement of RNAis mediated virus silencing, such as through enhanced vsiRNA production or stabilization of RNAi components; many of these observations were documented in transcriptomic and biochemical studies (Zheng et al., 2024). Implementation in practice must consider formulation, timing, and host genotype: e.g., low-doses of oligosaccharins can effectively prime strong antiviral immunity and fitness benefits, while a more holistic biocontrol management based on the biological agent must demonstrate persistence and compatibility with agronomic practices (Wang et al., 2024b).

### 8.5 Nano-formulations

Nanotechnology formulations are emerging tools that can effectively manage tobamovirus particles in the environment, as well as prime or boost the antiviral defenses of host plants (Eid et al., 2025). Nano-formulations limit tobamovirus infections by employing several distinct yet interconnected mechanisms. For example, metallic- and biopolymer-based nanoparticles can directly engage with virus particles and cause capsid disruption to prevent its entry and initial infection. NPs can also interfere with viral replication by binding viral RNA or replication proteins, while also promoting the accumulation of reactive oxygen species (ROS) that damage viral nucleic acids and proteins (Vasquez-Gutierrez et al., 2025). It provides a two-pronged approach that combines virucidal activity with resistance-induction. Recently, a multifunctional nano-protectant was documented to physically inactivate TMV particles in the environment while simultaneously enhancing plant immunity (Jiang et al., 2024). This study demonstrated that the nano-formulation reduced viral load and increased defense marker expression in treated plants. This provides strong evidence that engineered nanomaterials can couple the removal of extracellular viruses with immune priming. Additional supporting studies have indicated similar actions for metal and metal-oxide nanoparticles, such as zinc oxide (ZnO) and copper oxide (CuO) nanoparticles can bind and fragment TMV particles *in vitro*. They reduced their infectivity, and increasd plant defenses and PR gene expression when used as foliar or seed treatments (Abdelkhalek and Al-Askar, 2020).

The chitosan-based nano-formulation is of greater interest because of its elicitor properties. Chitosan nanoparticles and chitosan-nanocomposites offer boosted stability, slow and controlled release, more consistent ROS bursts, callose deposition, and SA pathway gene activation than bulk chitosan, leading to stronger reductions in viral accumulation across a range of pathosystems (Jaber et al., 2022). Silver (Ag) nanoparticles and biosynthesized Ag formulations have also shown to reduce TMV titers and delay replication. This is potentially due to factors linked to coat protein or nucleic acid interactions with the silver formulations, or associated with alterations of host secondary metabolism (e.g., flavonoid biosynthesis) (Sati et al., 2025). BioClay (dsRNA incorporated into layered double hydroxide, LDH) offers the sustained retention of dsRNA on the surface and a slow release of dsRNA, and protects sprayed plants from PMMoV for approximately 20–30 days (Mitter et al., 2017). Practical implementation includes research in the synthesis route, particle size, and coating mechanism, green synthesis (plant-extraction mediated), phytotoxicity and good viral treatment efficacy, and polymer coatings (chitosan, star-polycations) can stabilize nanoparticles and provide elicitor function (Jiang et al., 2024). It is important to note that lab and greenhouse research, while promising, is often heterogeneous. Therefore, before broad implementation, dose-response testing to establish standardization, alongside ecotoxicology evaluations and multi-season field testing. This should be combined with optimizing silver and chitosan formulations, resistant cultivars, and sanitation. Recent review conclude nano-encapsulated treatments to combat viruses indicate strong potential but require development and implementation within regulatory and safety limitations for sustainable use (Dutta et al., 2022).

## 9 Climate change and its impact on tobamovirus dynamics

High temperature influences viral activity by increasing MP activity and accelerating cell-to-cell transport in plants. As temperature rise, MP mobility increases due to accelerated ER–actin–myosin transport and enhanced plasmodesmal gating, leading to faster systemic spread. This suggests that tobamoviruses such as TMV, ToMV, and ToBRFV can proliferate more rapidly in warmer environments (Zheng et al., 2024). A critical concern is temperature-sensitive resistance. In Tomatoes, Tm-2²-mediated resistance is also disrupted above ~35 °C, allowing ToMMV and similar viruses to infect previously resistant genotypes. This susceptibility under heat stress indicates that traditional R-gene resistance may become ineffective in a warming climate.

Drought stress alters virus-to-host parameters. In TuMV–*Arabidopsis* models, viruses that evolved under drought conferred enhanced drought tolerance to infected plants. This indicates a potential shift towards mutualism under water-limiting conditions. Similarly, adaptive dynamics involving tobamovirus interactions may occur in drought-prone areas (González et al., 2021). Water deficit can also increase virus transmissibility. Experiments on TuMV and *cauliflower mosaic virus* under drought stress showed that aphid transmission rate of these viruses increased by 34-100%, despite no change in virus production. Mechanical or insect-transmission of tobamoviruses may also increase in water-stressed crops (Van Munster et al., 2017). Simultaneous exposure to multiple abiotic stressors has significantly impacted the plant immune system. *Arabidopsis* exposed to heat, drought, viral infection, transcriptomes show inhibition of R-gene associated defense mechanisms. The multifactorial stress caused a drastic decline in defense gene expression particularly TIR-NBS-LRR and salicylic acid pathway components, indicating that tobamovirus resistance can be lost to combined climatic stressors (Prasch and Sonnewald, 2013). The prevalence of viruses is also affected by seasonal and environmental cycles. In natural wild-host TuMV systems, viral abundance drops during cool seasons and increases during spring thaws. This suggests that changes in temperature lead to changes in the epidemiology of viruses. The incidence of tobamoviruses tends to peak during warm seasons, specifically in open-field tomato production (Van Munster et al., 2017; Moreno et al., 2022). These observations highlight the importance of developing pathogen-resistant crops with heat-tolerant R-genes, utilizing RNA-based sprays that are more effective in heat, and implementing more robust monitoring during heatwaves and droughts (Prakash et al., 2024).

## 10 Conclusion and future perspective

Tobamoviruses, particularly ToBRFV, remain among the most troublesome viruses to solanaceous crop production worldwide due to their unrivalled environmental stability, seed transmissibility and developing capacity to subvert conventional host resistance. The loss of durability in structurally stable genes like *Tm-2^2^*, among many others, indicates an urgent need for renewed activities in resistance breeding, development of new diagnostic techniques, and international collaboration in phytosanitary surveillance. Current developments in high-throughput sequencing, omics integration, and genome editing have significantly contributed to new insights into the epidemiology, evolution and plant-virus interaction of tobamoviruses. Such tools can track, enable early detection, and even generate specific resistance responses based on real-time virus-host dynamics. CRISPR-based diagnostics and interference platforms represent the future of virus and resistance detection. In addition to high specificity and sensitivity in detecting tobamoviruses, the CRISPR/Cas12 and Cas13 systems offer an opportunity to disrupt viral genomes in engineered crops (Rehman et al., 2024). Similarly, RNAi approaches targeting viral replication and movement gene have proven effective at alleviating systemic dissemination and symptoms severity, despite difficulties with stability in field conditions and off-target silencing (Borrelli et al., 2018). The development of small interfering RNAs (siRNAs) and artificial microRNAs (amiRNAs) targeting conserved regions of the tobamoviral genome could provide broad-spectrum resistance platforms for various crops. Omic integration of genomics, transcriptomics, proteomics, and metabolomics can provide a multilevel understanding of plant-virus interactions, stress responses, and the identification of early infection or resistance biomarkers.

Standardizing international seed-testing procedures and quarantine regulations will be critical in curbing outbreaks, especially given the involvement of international seed trade in the spread of tobamoviruses. Breeding programs would consider underexploited wild tomato relatives such as *Solanum chilense*, *S. pennellii*, and *S. peruvianum*, as sources of resistance to high temperatures and variable movement proteins. Moreover, interdisciplinary approaches combining virology, plant physiology, climatology, and bioinformatics are necessary to address all environmental drivers of tobamovirus emergence, including greenhouse crop cultivation, rising global temperatures, and increased seed transfer. Summing up the development of the battle against tobamoviruses, the most prominent trend in the field is the combination of molecular innovation, ecological knowledge, and regulatory coordination. It will be essential to invest further in host pathogen genomics, precision breeding, and scalable diagnostic infrastructure to ensure crop productivity and biosecurity in a world of constant flux.
